# Population Pharmacokinetic Model of Iohexol in Dogs to Estimate Glomerular Filtration Rate and Optimize Sampling Time

**DOI:** 10.3389/fphar.2021.634404

**Published:** 2021-04-29

**Authors:** Sarah Baklouti, Didier Concordet, Vitaliano Borromeo, Paola Pocar, Paola Scarpa, Petra Cagnardi

**Affiliations:** ^1^ INTHERES, Université de Toulouse, INRAE, ENVT, Toulouse, France; ^2^ Laboratoire de Pharmacocinétique et Toxicologie, CHU de Toulouse, Toulouse, France; ^3^ Department of Veterinary Medicine, Università Degli Studi di Milano, Milano, Italy

**Keywords:** dog, population pharmacokinetic, iohexol plasma clearance, sampling time optimization, glomerular filtration rate estimation

## Abstract

Monitoring iohexol plasma clearance is considered a useful, reliable, and sensitive tool to establish glomerular filtration rate (GFR) and early stages of kidney disease in both humans and veterinary medicine. The assessment of GFR based on iohexol plasma clearance needs repeated blood sampling over hours, which is not easily attainable in a clinical setting. The study aimed to build a population pharmacokinetic (Pop PK) model to estimate iohexol plasma clearance in a population of dogs and based on this model, to indicate the best sampling times that enable a precise clearance estimation using a low number of samples. A Pop PK model was developed based on 5 iohexol plasma samples taken from 5 to 180 minutes (min) after an intravenous iohexol nominal dose of 64.7 mg/kg from 49 client-owned dogs of different breeds, sexes, ages, body weights, and clinical conditions (healthy or presenting chronic kidney disease CKD). The design of the best sampling times could contain either 1 or 2 or 3 sampling times. These were discretized with a step of 30 min between 30 and 180 min. A two-compartment Pop PK model best fitted the data; creatinine and kidney status were the covariates included in the model to explain a part of clearance variability. When 1 sample was available, 90 or 120 min were the best sampling times to assess clearance for healthy dogs with a low creatinine value. Whereas for dogs with CKD and medium creatinine value, the best sampling time was 150 or 180 min, for CKD dogs with a high creatinine value, it was 180 min. If 2 or 3 samples were available, several sampling times were possible. The method to define the best sampling times could be used with other Pop PK models as long as it is representative of the patient population and once the model is built, the use of individualized sampling times for each patient allows to precisely estimate the GFR.

## Introduction

Glomerular filtration rate (GFR) is considered the most useful indicator of the overall kidney function and a sensitive biomarker to establish early stages of kidney disease in both humans and veterinary medicine (Schwartz et al., 2006; Lefebvre 2011). In current practice, GFR is generally determined by different methods monitoring plasma clearance of a marker substance, and iohexol is considered a useful and reliable plasma clearance marker (Delanaye et al., 2016b). It is an iodinated non-radiolabeled radiographic contrast agent with low extrarenal excretion, low protein binding, and is neither secreted nor reabsorbed by the kidney (Delanaye et al., 2016a). When used for clearance studies, iohexol has nearly no toxicity and is commercially available at a very low cost. Thus, it has become a key tool to measure GFR (Nilsson-Ehle 2001; Benz-de Bretagne et al., 2012; Gaspari et al., 2018) or even a gold standard (Åsberg et al., 2020) both in human and in veterinary medicine (Gleadhill and Michell 1996; Heiene and Moe 1998; Finco et al., 2001; Goy-Thollot et al., 2006; Bexfield et al., 2008; Lippi et al., 2019a; Lippi et al., 2019b; Pocar et al., 2019).

The assessment of GFR based on iohexol plasma clearance has a major limitation on the requirement of repeated blood sampling over several hours, which is not easily feasible in a clinical setting. Thus, strategies to reduce the number of sampling have been exploited as first reported by Bröchner-Mortensen (1972) in humans. All of these foresaw the addition of correction formulas to achieve more accurate GFR estimation (Gleadhill and Michell 1996; Bexfield et al., 2008; Von Hendy-Willson and Pressler 2011; Sasaki et al., 2015; Pocar et al., 2019). Nevertheless, from veterinary practitioners there is still a demand for reliable and easily applicable GFR estimations methods in a clinical setting.

Population pharmacokinetics (Pop PK) has been developed to assess the sources of variability of pharmacological agents PK disposition in a target population, and thus to define and estimate the impact of specific factors, such as demographic, pathophysiological, environmental, and drug related (Ette and Williams 2004; Kiang et al., 2012). Additionally, it is able to limit the requirements of sampling designs by applying mixed-effects modeling approaches (Taubert et al., 2018). Moreover, Pop PK approaches are useful to predict a typical PK profile for any given patient when a sufficient knowledge of covariates is available (Concordet et al., 2004).

Starting from recently published data from the same group of authors (Pocar et al., 2019), the first aim of this work was to build a Pop PK model to estimate iohexol plasma clearance for GFR assessment in a population of dogs. Based on this model, the second aim was to indicate the best sampling times that enable a precise clearance estimation using a limited number of samples.

## Materials and Methods

### Animals

With ethical approval (Organismo Preposto al Benessere Animale, OPBA_107_2016) and after written consent by the owners, 49 client-owned dogs were enrolled for the study. Dogs were of different breeds, sexes, ages, body weights, and clinical conditions (healthy or presenting chronic kidney disease, CKD), all scheduled at the University Veterinary Teaching Hospital of the University of Milan for different clinical procedures. A complete physical examination was performed on each dog shortly before GFR evaluation together with complete blood count, serum biochemistry profile and routine urinalysis, UPC ratio, and ultrasound examination. According to the guidelines of the International Renal Interest Society (IRIS), dogs were defined as healthy (CKD−, negative) or were diagnosed with chronic kidney disease (CKD+, positive) (Polzin et al., 2005).

### Sample Collection and Analysis

The whole sampling protocol and analysis were fully described in Pocar et al. (2019). Briefly, iohexol was administered over 60 s periods as intravenous (IV) bolus injection via the catheter placed in the left cephalic vein at the nominal dose of 64.7 mg/kg. Blood samples were obtained from the right cephalic vein at 5, 15, 60, 90, and 180 min after administration of the marker, placed in a heparinized tube, and centrifuged to obtain plasma. Samples were stored at −40°C until extraction and iohexol quantification by a validated HPLC method as reported by Pocar et al. (2019). Details on samplings, methods for iohexol determination, and GFR estimation described in Pocar et al. (2019) are summarized in Supplementary File S1.

### Population Pharmacokinetics

Pharmacokinetic modeling was carried out using commercially available software (MONOLIX® version 2018R2, Lixoft, Antony, France). A nonlinear mixed effects (NLME) approach was used to generate Pop PK parameter estimates, θ, the interindividual variability, Ω, and the residual variability, σ^2^. One-, two-, and three-compartment models were evaluated to identify the model that best described the dataset. Model selection was carried out according to different criteria: 1) visual inspection of different diagnostic plot (i.e., visual predictive check and residual plot), 2) precision of the estimated parameters, 3) correct estimation of the information fisher matrix, and 4) values of the objective function (i.e., likelihood ratio test, LRT; Akaike information criterion, AIC; and Bayesian information criterion, BIC). The constructed model was of the form:

$$\left\{ \begin{array}{l} \varphi_{i}=A_{i}\theta \exp\left(\eta_{i}\right),\;\mathrm{where}\eta_{i}∼\mathbb{N}\left(0,\Omega \right),\\ Y_{ij}=f\left(t_{ij};\;\varphi_{i}\right)+g\left(t_{ij};\varphi_{i};b\right)\varepsilon_{ij},\;\mathrm{where},\varepsilon_{ij}∼\mathbb{N}\left(0,1\right). \end{array}\right.$$

Here, *φ*_*i*_ is a vector containing individual pharmacokinetic parameters for the i^th^ individual (or its natural logarithm, the vector *lnφ*_i_). *Y*_*ij*_ is the j^th^ concentration performed on the individual *i* at the moment *t_i_*_*j*_, *ε* *_ij_* is the j^th^ residual error for the individual *i* at the moment *t_i_* *_j_*, *f* is a function describing structural model (i.e., it describes concentration evolution over time), *g* is a function describing the residual error model, *A*_*i*_ is a known matrix including covariates of the i^th^ individual, *θ* is a fixed effects vector including population parameters, Ω is the *η*_*i*_ 's variance-covariance matrix, *b* is the vector of the parameters involved in the residual error model, and *η*_*i*_ is the vector of the random effects involved in interindividual variability for the i^th^ individual.

Covariate analysis was performed and the best covariate model was selected based on the decrease of the unexplained interindividual variability, the improvement of the objective function, and the absence of precision's loss in the estimated parameters. Nine covariates were tested on individual PK parameters taking into account their plausibility and plots such as individual parameter vs. covariates. Weight, age, creatinine, urea, and urine-specific gravity (USG) were considered as continuous covariates, whereas kidney status (healthy: CKD− and diseased: CKD+), gender, and breed as categorical covariates. These covariates contain the available information in each dog other than the iohexol concentrations.

### Optimal Sampling Time

Based on the Pop PK model, we looked for the sampling times that allowed the best estimation of iohexol clearance, and therefore the GFR for each dog. These “best sampling” times depend on the available relevant information collected in the dog, that is, the covariates retained in the final Pop PK model.

We tried to estimate GFR using 1 single sampling time or 2 or 3 sampling times. These were looked for between 30 and 180 min discretized with a step of 30 min. T1, T2, and T3 were defined as the sets of tested combinations of 1, 2, or 3 times, respectively (*i.e.,* T1 = {30;…180}; T2 = {(30, 60); … (150, 180)}; T3 = {(30, 60, 90); … (120, 150, 180)}).

The best sampling times change from an individual to another according to the covariates values, thus, we chose to give three examples of sampling times determination according to the kidney status and creatinine concentration, defined as follows: 1) CKD− with a creatinine value equal to 0.98 mg/dl; 2) CKD+ with a creatinine value equal to 1.7 mg/dl; 3) CKD+with a creatinine value equal to 2.25 mg/dl. These three creatinine values, low, medium, and high, were chosen to illustrate the performances of our approach and represent different renal functionalities.

The criterion we chose to compare (and thus select) the different combinations of sampling times was the mean square error (MSE) for the estimation of GFR. The MSE written as MSE = bias^2^+SD_imprecision_^2^ is a kind of proxy statistics that balances the bias and the imprecision of estimation.

Using the population parameters previously estimated, 5,000 theoretical pharmacokinetic profiles were simulated for each dog for which the GFR was to be estimated. In other terms, 5,000 profiles were simulated for a combination of covariates *A*. For a given dog, whose covariates values were contained in *A*, the simulated concentrations were obtained using the following model:

{φi∗=Aθ^exp(ηi∗), where ηi∗∼ℕ(0,Ω^),i=1,…,5000,Yit=f(t ; φi∗)+g(t;φi∗;b^)εij∗, where εij∗∼ℕ(0,1),

and t∈K⊂T1 or T2 or T3.

Then, for each simulated PK profile and each combination *K* of 1, 2, or 3 sampling times, the corresponding simulated concentrations were used to predict the GFR. It appeared in the Pop PK model as a component of *φ.*

For a given individual, the empirical Bayes estimates (EBE) is a classical predictor of its individual PK parameters *φ*. It is obtained as $\hat{\varphi_{K}}=h\left(\hat{\eta_{K}},\hat{\theta },A\right)$ where

$$\hat{\eta_{K}}=\arg\sup\;P\left(\eta |Y_{K}\right).$$ (1)

More precisely, if we work on the simulated concentrations *Y* ^∗^_*i,t*_

$$\hat{\eta_{i,K}^{*}}=\arg\inf_{\eta }\sum_{t\in K}\frac{{\left(Y_{i,t}^{*}-f\left(t,h\left(\eta ,\hat{\theta },A\right)\right)\right)}^{2}}{g^{2}(t,h\left(\eta ,\hat{\theta }\;,A\right),\hat{b})}+\ln g^{2}\left(t,h\left(\eta ,\hat{\theta },A\right),\hat{b}\right)\;+\eta^{\prime }{\hat{\Omega }}^{-1}\eta .$$ (2)

A Gauss Newton algorithm can be used to minimize this equation.

For the individual for whom the GFR was to be predicted and for a fixed combination of times *K*, we computed the 5000 EBE's ${\left(\hat{\eta_{i,K}^{*}}\right)}_{i=1,\ldots ,5000}$ using the simulated concentrations obtained with the same covariate values as the individual of interest by minimizing Eq. 2. The distance between these EBE's and the actual ηi* used to simulate the concentrations gave information on the performances that could be achieved by estimating the GFR using such a choice of sampling times *K*. This distance could be evaluated by the mean square error (MSE) defined as:

$$MSE_{K}=\frac{1}{5000}\sqrt{\sum_{i=1}^{5000}{\left(\hat{\eta_{i,\;\;K}^{*}}-\eta_{i}^{*}\right)}^{2}}.$$

*MSE*_*K*_ represented the average distance (in log-scale) between the simulated clearance (i.e., clearance obtained by Monte Carlo simulations using the Pop PK model) and the estimated clearance. The combination of times *K* with the smallest *MSE*_*K*_ were, on average, the best combination of times that can be used to estimate the GFR of the dog.

The GFR estimates with EBE were also compared to the GFR obtained by a non-compartmental approach in the previous study by Pocar et al. (2019) that consists of computing Dose/AUC/BW (Supplementary File S1). In this formula, BW is the bodyweight of the animal and AUC is the area under the iohexol concentration's curve *vs.* time. This AUC should be determined from 0 (time of the iohexol administration) to infinity. Because it was expected that no concentrations were small at the last sampling time (180 min), the non-observed part of the curve (post 180 min) was extrapolated to compute the AUC. A Bland–Altman plot was used to compare the results of our approach and the previous one.

## Results

### Animals and Iohexol Concentrations

**FIGURE 1:**
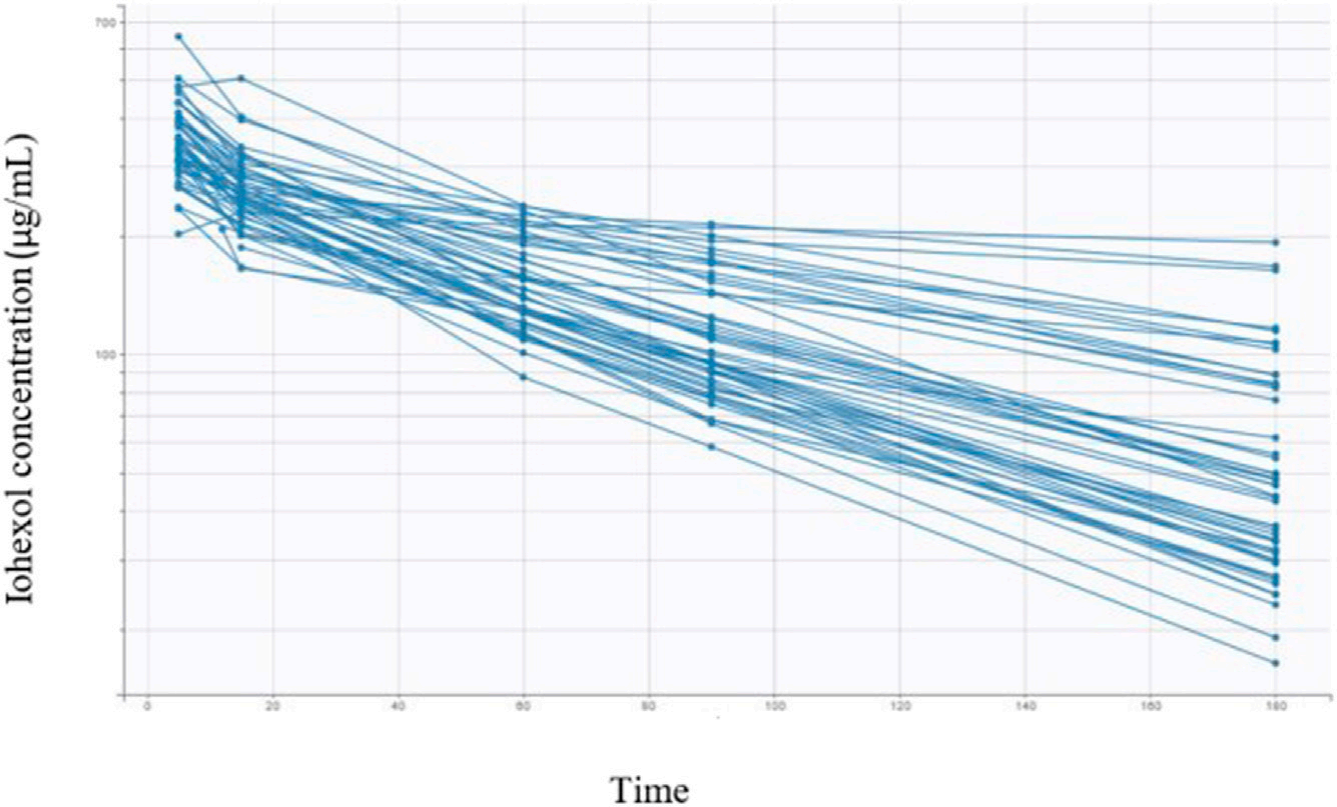
Semi-logarithmic spaghetti plots of iohexol plasma concentrations over 180 min after a single i.v. administration (nominal dose of 64.7 mg/kg) in 49 dogs.

All animal characteristics are reported in Table 1 together with the covariates and coding used for Pop PK modeling. Twenty nine dogs were healthy and 20 diagnosed positive for CKD. The dogs’ breeds were heterogeneous, with 10 mongrels and 39 dogs representing 21 different pure breeds. A total of 245 blood samples were taken from all dogs (*n*. 49) at time intervals of 5, 15, 30, 60, 90, and 180 min. The measured iohexol concentrations ranged from 16.4 μg/ml to 643.6 μg/ml. Iohexol plasma concentrations obtained in all 49 dogs are reported in Figure 1.

**TABLE 1:** Animal characteristics, covariates, and coding used in Pop PK analysis.

Continuous covariates			
	Mean ± S.D.	Range (median)	
Age (y)	5.43 ± 3.5	0.4–16 (4.5)	
Body weight (kg)	25.8 ± 9.5	3.9–46 (27.6)	
Serum creatinine (mg/dl)	1.47 ± 1.99	0.67–14.4 (1.09)	
Serum urea (mg/dl)	44.95 ± 34.81	16–181.4 (36)	
Urine specific gravity (USG)	1,036.78 ± 18.59	1,003–1,065 (1,040)	
Categorical covariates			
	Type and number of subjects (code)		
Renal status (chronic kidney disease, CKD)	Healthy CKD− *n* = 29 (Code 0)	Diseased CKD + *n* = 20 (Code 1)	
Breed	Mongrel *n* = 10 (Code 0)	Other breeds *n* = 39 (Code 1)	
Sex	Male *n* = 19 (Code 0)	Female *n* = 6 (Code 1)	Female neutered *n* = 24 (Code 2)

### Population Pharmacokinetics

**FIGURE 2:**
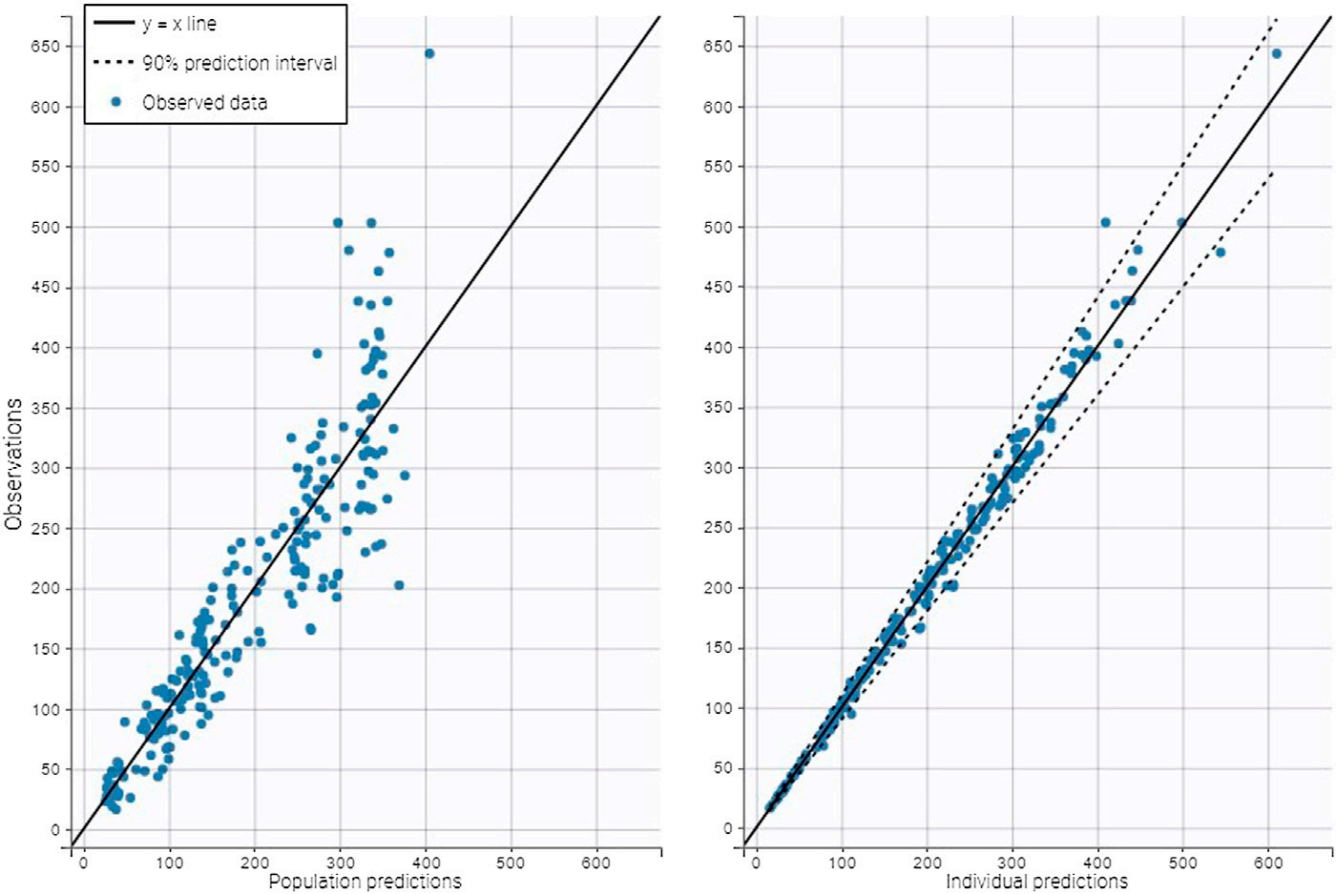
Plots of the observed iohexol concentration (µg/ml) vs. population predicted concentration **(left)** or vs. individual predicted concentration **(right)**. The points are distributed homogeneously around the identity line showing the model well described the data.

**FIGURE 3:**
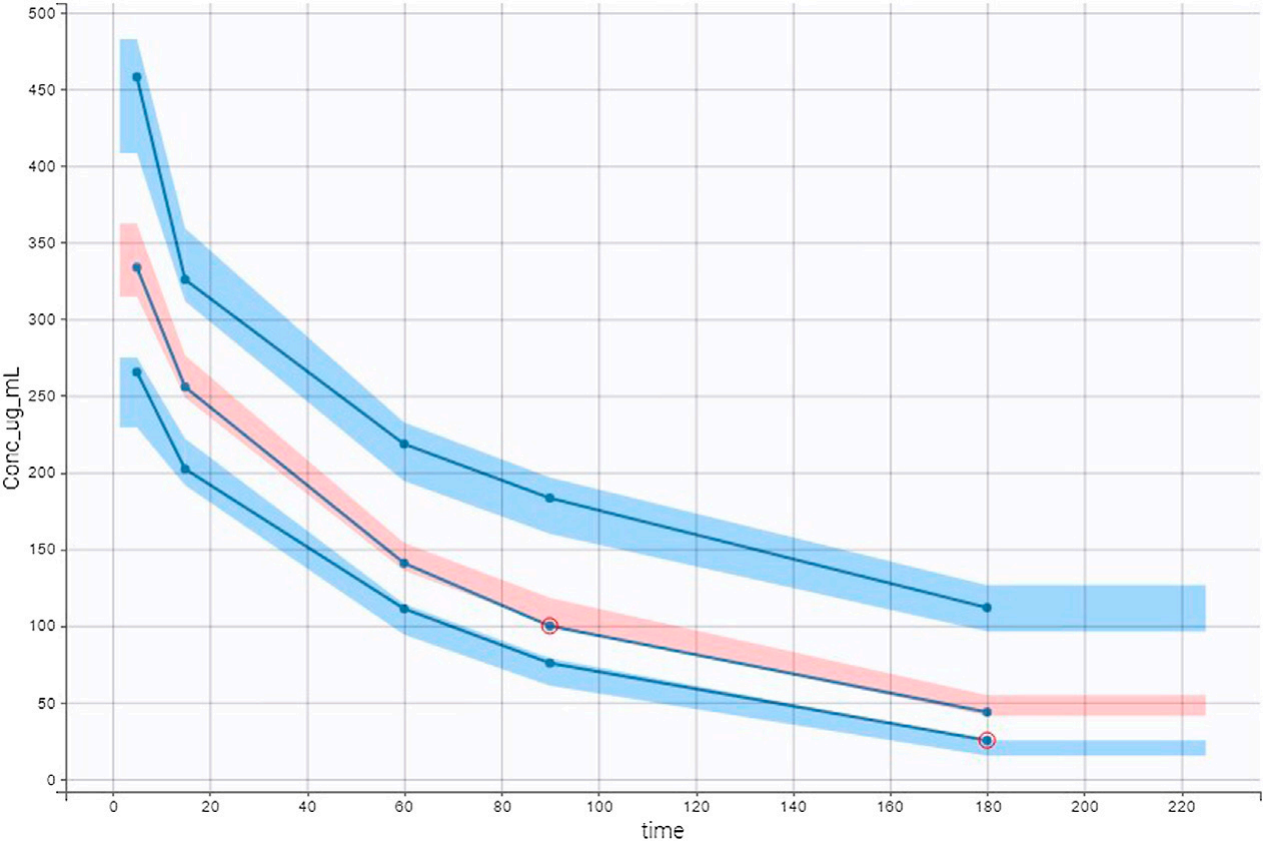
Visual predictive check (VPC) plot was obtained with empirical data (blue lines) and simulated data. Multiple Monte Carlo simulations allowed defining theoretical percentiles (10^th^, 50^th^, and 90^th^) and their prediction interval. Empirical and theoretical percentiles are superposed showing that the model well described the data.

A two-compartment model best fitted the data, as shown in the plots of the observed iohexol concentration vs. population predicted concentration or vs. individual predicted concentration (Figure 2) and in the visual predictive check plot (Figure 3). Population value for clearance (*θ*_*Cl*_), central volume of distribution (*θ*_*V*__*1*_), peripheral volume of distribution (*θ*_*V*__*2*_), and intercompartmental clearance *θ*_*Q*_ were estimated and the model included interindividual variability (IIV) on *θ*_*Cl*_, *θ*_*V1*_, and *θ*_*V2*_ (Table 2). The variability error model was best described by a proportional error *є* (Table 2). The value of the residual error was 6.17%. The correlations between individual parameters were low (<30%) and were therefore not included in the final Pop Pk model.

**TABLE 2:** Synthesis of estimates obtained in the final Pop PK model.

Parameters	Units	Value	SE	RSE (%)
*θ_Cl_*	L/min/kg	0.00212	0.00010	4.68
*θ_V1_*	L/kg	0.163	0.00661	4.07
*θ_V2_*	L/kg	0.058	0.00387	6.64
*θ_Q_*	L/min/kg	0.0034	0.00042	12.21
Covariates				
θ1 (diseased dogs)		−0.379	0.07002	18.49
θ2 (creatinine)	dl/mg	−0.421	0.05356	12.72
Variability				
*ηC*1		0.208	0.02255	10.85
*ηV*1		0.248	0.02718	10.95
*ηV2*		0.199	0.05618	28.21
Residual error				
*ℇ*		0.0617		

Two covariates were included in the final model. They allowed explaining a part of clearance variability. These covariates were centered creatinine value and kidney status defined according to the IRIS score. Covariates were added according to an exponential model. The following equation was used as a covariate model:

$$\text{ln}\left(Cl\right)=\text{ln}\left(\theta_{Cl}\right)+\theta_{1}*1_{\left[\text{diseased dogs}\right]}+\theta_{2*\text{ccreatinine }\left(\text{mg}/\text{dL}\right)+\eta Cl}.$$

Here 1_[diseased dogs]_ = 1 for dogs with diseased kidney status (CKD+) and 0 for dogs with healthy kidney status (CKD−) and *ccreatinine* is the creatinine centered around an average value.

### Optimal Sampling Time

**FIGURE 4:**
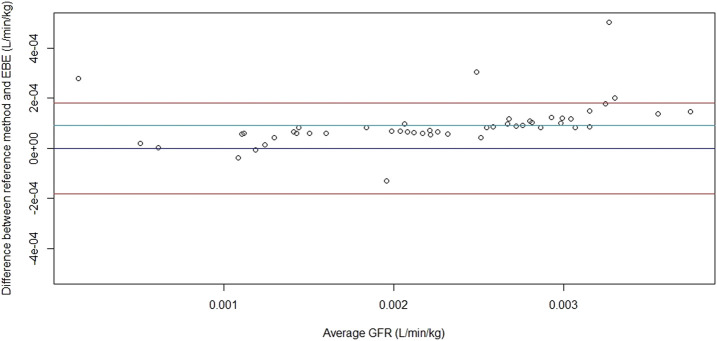
The Bland–Altman plot of the comparison of GFR obtained with the formula Cl = D/AUC in the previously published study by Pocar et al. (2019) and empirical Bayes estimates (EBE) (five sampling times available).

Figure 4 shows the Bland–Altman plot comparing the GFR obtained by our method to the one obtained by Pocar et al. (2019) when using all the available concentrations. On the *x*-axis, the means of the two methods are represented (i.e., reference method by Pocar et al., 2019 and our method based on EBE). On the *y*-axis, the differences between the two methods are shown. The red line depends on the standard error and represents imprecision. The light blue line represents the mean and therefore the bias. The figure shows that the performances of the GFR estimation with the previous and our method are the same when all the available times are used.

**FIGURE 5:**
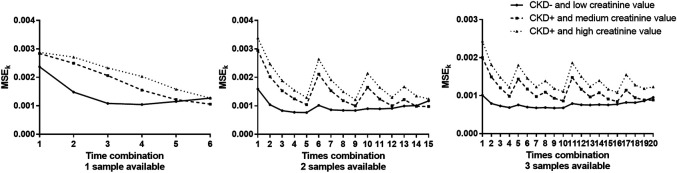
Plots of mean square error for the time combination K (MSE_K_) vs. time for the 3 dog examples (CKD− and low creatinine value, CKD+ and medium creatinine value and CKD+ and high creatinine) when 1, 2, or 3 samples were available (left, middle, and right, respectively ).

MSE_K_ results are plotted in Figure 5 and are summarized in Tables 3–5 for the three examples values. The best sampling time depended on the health status and the number of possible samples. When 1 sampling time was available, the best times for clearance calculation were 90 or 120 min for example 1 dogs (CKD− and creatinine value = 0.98 mg/dl), 150 or 180 min for example 2 dogs (CKD+ and creatinine value = 1.7 mg/dl), and 180 min for example 3 dogs (CKD+ and creatinine = 2.25 mg/dl). When 2 sampling times were available, several sampling times were possible for the three dog examples. Note that for dogs CKD+ and with a high creatinine value (example 3), all the recommended time combinations included sampling at 180 min. When 3 sampling times were available, several times combinations could be envisaged for dogs of examples 2 and 3, whereas for dogs CKD− and with a low creatinine value (example 1) all combinations gave similar results, except the combination of the three initial sampling times (30, 60, and 90 min).

**TABLE 3:** Mean square error for the time combination K (MSE_K_) results of optimal designs including 1 sample (TC: time combination). The best sampling times for each example of dogs are in red.

		Example 1 (CKD− and creatinine value = 0.98 mg/dl) (x10^−3^)	Example 2 (CKD+ and creatinine value = 1.7 mg/dl) (x10^−3^)	Example 3 (CKD+ and creatinine value = 2.25 mg/dl) (x10^−3^)
TC	1 sampling time	MSE	MSE	MSE
1	30	2.37144	2.83923	2.87982
2	60	1.48213	2.49541	2.71283
3	90	1.08587	2.06094	2.33112
4	120	1.04398	1.55052	2.03204
5	150	1.15137	1.21796	1.58001
6	180	1.27023	1.05704	1.27007

**TABLE 4:** Mean square error for the time combination K (MSE_K_) results of optimal designs including 2 samples (TC: times combination). The best sampling times for each example of dogs are in red.

		Example 1 (CKD− and creatinine value = 0.98 mg/dl) (x10^−3^)	Example 2 (CKD+ and creatinine value = 1.7 mg/dl) (x10^−3^)	Example 3 (CKD+ and creatinine value = 2.25 mg/dl) (x10^−3^)
TC	2 sampling times (min)	MSE	MSE	MSE
1	30_60	1.59044	2.94597	3.38164
2	30_90	1.04184	2.02553	2.47435
3	30_120	0.8313	1.52744	1.88487
4	30_150	0.77656	1.24404	1.54674
5	30_180	0.76386	1.04572	1.29145
6	60_90	1.01744	2.11078	2.63993
7	60_120	0.8628	1.53112	1.91978
8	60_150	0.84146	1.18311	1.49829
9	60_180	0.83782	1.00331	1.21676
10	90_120	0.90116	1.65281	2.14052
11	90_150	0.89505	1.23837	1.6483
12	90_180	0.91048	1.00323	1.29749
13	120_150	0.99535	1.22089	1.67092
14	120_180	1.00764	0.9865	1.34951
15	150_180	1.17352	0.97719	1.24635

**TABLE 5:** Mean square error for the time combination K (MSE_K_) results of optimal designs including 3 samples (TC: times combination). The best sampling times for each example of dogs are in red.

		Example 1 (CKD− and creatinine value = 0.98 mg/dl) (x10^−3^)	Example 2 (CKD+ and creatinine value = 1.7 mg/dl) (x10^−3^)	Example 3 (CKD+ and creatinine value = 2.25 mg/dl) (x10^−3^)
TC	3 sampling times (min)	MSE	MSE	MSE
1	30_60_90	1.01127	2.00212	2.43594
2	30_60_120	0.79361	1.49464	1.81317
3	30_60_150	0.72799	1.20321	1.48077
4	30_60_180	0.68501	0.98346	1.22154
5	30_90_120	0.75675	1.43671	1.80194
6	30_90_150	0.69878	1.17373	1.45919
7	30_90_180	0.67861	0.97652	1.2322
8	30_120_150	0.68581	1.09122	1.38739
9	30_120_180	0.67313	0.93237	1.18603
10	30_150_180	0.67858	0.8623	1.10814
11	60_90_120	0.79057	1.47998	1.86321
12	60_90_150	0.75929	1.1665	1.50283
13	60_90_180	0.75331	0.96534	1.23779
14	60_120_150	0.76469	1.07649	1.396
15	60_120_180	0.75608	0.9122	1.16991
16	60_150_180	0.77419	0.84264	1.08415
17	90_120_150	0.82071	1.14447	1.55208
18	90_120_180	0.81691	0.94613	1.2775
19	90_150_180	0.86003	0.88547	1.18735
20	120_150_180	0.95395	0.884564	1.23236

## Discussion

To the author’s knowledge, this is the first study that builds a Pop PK model to estimate iohexol plasma clearance in a population of dogs and by the model, an innovative approach is reported to choose the best sampling time to precisely estimate iohexol clearance and therefore GFR with a reduced number of samples. The study is innovative and useful, considering the constant demand for reliable and easily applicable GFR estimations methods by veterinary practitioners. Nevertheless, the approach and model here reported could be considered a “model” for studies in other species and humans.

No other studies report a Pop PK model to estimate clearance and GFR in dogs, but in humans a recent study by Taubert et al. (2018), reports a Pop PK model using a three-compartmental model to better estimate iohexol clearance. These authors support the existence of a third compartment where iohexol rapidly distributes soon after administration and affects the whole concentration–time curve. More recently, Åsberg et al., 2020 reported a Pop PK method to determine iohexol clearance in humans using a two-compartment model.

Similarly, the PK parameters of iohexol in dogs were best described by a two-compartment model with a linear elimination. The different sampling times of our study (till 180 vs. 300 min by Taubert et al., 2018) can have influenced the detection of a third compartment, so as the old age of the patients (>70 years) in Taubert et al., 2018 study. Moreover, to support our model, the interindividual variability was around 20% on all the parameters except intercompartmental clearance that was fixed. Furthermore, all parameters in the model were precisely estimated with percent relative standard errors <15% for the fixed effect and <30% for the standard deviation of the random effects (Table 2). None of the goodness-of-fit plots showed a systematic bias or trend. The intraindividual variability was around 6%, which is small enough to expect precise Bayesian estimations.

The robustness of the results was strengthened by the inclusion of a varied population of dogs both by their demographical characteristics and by their renal status. Indeed, dogs’ weights ranged from 3.9 kg to 46 kg. The values of creatinine and urea varied, respectively, from 0.67 to 14.4 mg/dl and from 16 to 181.4 mg/dl. Finally, female, male, and female neutered dogs were included in the study allowing to study a sex-effect. Furthermore, the Pop PK parameters' estimates were consistent with the data published in our previous work (Pocar et al., 2019) and this supports the reliability of our Pop PK model.

Covariate analysis was performed to identify the influence of various baseline characteristics on the PK of iohexol. Two covariates explained a part of the variability of iohexol elimination clearance: plasma creatinine and kidney status (CKD− or +). The creatinine value as a major covariate was not surprising as both creatinine and iohexol are eliminated by glomerular filtration. The kidney status (CKD− or +) that takes into account of the creatinine value and different clinical and biological variables was also a significant covariate of iohexol elimination clearance. Urea could have been an interesting covariate as its elimination mechanism is mainly by glomerular filtration. Nevertheless, in this study, it did not appear to explain a part of the elimination clearance variability of iohexol and this could be explained by two reasons. On one hand, urea is less specific than creatinine to indicate kidney disease (Finco and Duncan, 1976). On the other hand, the part of variability on the elimination clearance explained by urea is the same as the one explained by creatinine. Thus, urea appears as a covariate only in the absence of data on creatinine.

Otherwise, the covariate analysis did not reveal the influence of any breed or age on the iohexol PK. Physiologically, age was expected to be a significant covariate due to the progressive decrease of the glomerular filtration rate with age. However, here it did not appear as an influential covariate probably because the age cannot be interpreted without the breed or body size (Kraus et al., 2013). To put in evidence an effect of age, we should have related age to life expectancy. Moreover, the breeds represented in this study were too heterogeneous (mongrels and 21 different pure breeds) to influence the model.

To cope with the need for reliable and feasible GFR estimation methods in the clinical practice, we looked for a method to reduce the number of necessary samples to precisely estimate iohexol clearance, and therefore the GFR, in order to promote its use in the clinics. It was not the definition of an optimal design, which, by definition, requires as many sampling times as the number of individual PK parameters to estimate. Therefore, after obtaining the model, we studied a methodology to obtain the best sampling times to allow a correct estimation of the iohexol clearance. This methodology was different from the previous methodology already published that used the D optimality and Ds optimality criterion (Mentré et al., 1997; Tod et al., 1998; Fedorov, 2013). Indeed, these criteria focused on determining the best sampling times that minimize the asymptotic evaluation of imprecision of the individual clearance. This approach is relevant when the number of blood sampling performed in the individual under investigation is large. Differently, we wanted to estimate parameters with a maximum of 3 blood samples, but our estimate could be both biased (eta-shrinkage) and imprecise. This was the reason why we used the MSE that combines both imprecision (variance) and bias, and indicates how close the estimator is to the true value (Walther and Moore, 2005).

Elimination clearance was larger in dogs CKD− and with low creatinine value (example 1) than in dogs CKD+ (examples 2 and 3). It was thus expected that the best 1 sampling time for animals CKD− and with a low creatinine value (example 1) was earlier than the one for the other animals. In fact, in our study when experimental design with 1 sampling was considered, the best sampling times were 90 or 120 min for dogs CKD−with the low creatinine value (example 1) and 180 min for dogs CKD+ with the high creatinine value (example 3). Surprisingly, the optimal sampling time for dogs with a creatinine value equal to 1.7 mg/dl was the same as for dogs with a creatinine value equal to 2.25 mg/dl (150 or 180 min). It was probably because the last sampling time (180 min) was too early for sick dogs. In fact, all dogs CKD+ with the high creatinine value had an iohexol concentration greater than 100 μg/ml at 180 min. These concentration values represented more than 50% of the peak concentration (taken 5 min after the injection) except for one dog for which the 180 min concentration represented 20% of the 5 min concentration. These results showed that the elimination was not ended and that the real optimal experimental design might include times later than 180 min for the sick dogs. These results could also call into question the quality of the parameters estimates provided by the model, especially clearance, but this was not the case, because the distribution of iohexol is very rapid since it diffuses freely through membranes due to its chemical properties. It has been demonstrated that in CKD+ dogs the slope of the iohexol plasma decay curve decreases at three hours (180 min) leading to the formation of an excretion plateau (Lippi et al., 2008). The same authors observed that estimated GFR based on a sampling protocol ending at 180 min shows better correlation with real GFR than protocols including later timing (e.g., 300 or 420 min). Finally, single sampling protocols at 180 min for GFR estimation have been demonstrated to have an acceptable error margin for both healthy and sick dogs (Pocar et al., 2019). Considering the clinical practice, it is important to have a reliable evaluation of iohexol clearance and consequently GFR as soon as possible and within a reasonable time when patients are in the hospital. Thus, although iohexol elimination has not ended, a 180 min sampling time is a useful tool to help the clinicians in detecting kidney disease resulting in the best compromise between the accuracy of GRF estimation and the time advantage for the dog and owner.

When 2 or 3 sampling times were considered, for the dogs CKD+ with medium or high creatinine values (example 2 and 3), the best results were obtained from the combination of times including times far from the administration, as also observed in human transplanted patients, where the optimal combination of time points included concentrations measured at the extreme times (120 and 270 min) (Benz de-Bretagne et al., 2012). For dogs CKD− and with a low creatinine value, the results were more smoothed and many combination times could be considered especially with 3 sampling times, where all except the first can be considered. For these dogs, MSE was not improved when 1, 2, or 3 sampling times were considered, thus, 1 well-chosen sampling time is sufficient to precisely estimate clearance and taking other samples is not necessary. The same observation can be done also for dogs CKD+ and medium or high creatinine values, as, also for these dogs, MSE did not change when combining 1, 2, or 3 sampling times. In summary, before estimating GFR in a dog using iohexol, knowing the creatinine value is essential to choose the best sampling time and to limit costs and save time. Then, the methods to further estimate GFR based on iohexol clearance and limited sampling times can follow the different steps reported by Pocar et al. (2019).

The method to define the best sampling times could be used with other Pop PK models as long as the model is correctly built, includes covariates to limit the interindividual variability and presents a weak value for the intraindividual variability.

The limited number of dogs in this study may have had an impact on our results for three main reasons: 1) The sample size is directly linked to the representativeness of the sample and thus to the possibility to extrapolate the results to other dogs. As already mentioned, the aim of this article was to use all available and relevant information in a dog to estimate its GFR. We found that plasma creatinine and renal status based on the IRIS score gave information on GFR, but it is not clear if the relationship between GFR and the creatinine/renal status is the same for all dog breeds. A study with only 49 dogs cannot be representative of all dogs breeds, as there exists more than 300 dog breeds. Thus, a study with several thousands of dogs would be necessary to properly document this. 2) The sample size is also limited for the validation of the Pop PK model. While we strictly validated the model according to the recommendations given in the best PK journals for Pop PK model validation, we agree, in view of point 1) that this sample size is not large enough for a population validation. We rely on the results given for the breeds represented in this study, but we cannot exclude that these results would be approximate for other breeds. 3) The article wants to propose a methodology to choose the best sampling times to estimate a dog GFR according to the information available in this dog. The sampling times were chosen so that the MSE was minimal. The sample size has a direct influence on the imprecision of the MSE (MSE = bias^2^+SD_imprecision_^2^) estimation; that is, on the bias and imprecision estimation. To our knowledge, no methods are allowing to estimate *a priori* (before having any preliminary results) the sample size for bias and imprecision estimation in such models. The best published results on this topic (Mentré et al., 1997; Tod et al., 1998; Fedorov 2013; Mentré et al., 2013) are asymptotic (one assumes that the sample size is infinite) and require precise knowledge of the model parameters. Even if the sample size is important for the MSE estimation, the "optimal sampling" times obtained with an imprecise MSE can be expected to be close to the one that would be obtained with larger sample size.

Finally, this work supports a global approach of personalized medicine and its application in human medicine or other veterinary species can be possible, like for example in cats. Cats are low compliant patients and reducing sampling numbers can greatly reduce the animal stress and could make the GFR procedure reasonably feasible in the clinical practice. The only prerequisite of this methodology is to obtain data sufficiently reliable to build a Pop PK model representative of the patient population. Once the model is built, the use of individualized sampling times for each patient allows estimating with precision the GFR.

## Data Availability

The original contributions presented in the study are included in the article/Supplementary Material, further inquiries can be directed to the corresponding author.
