# Supplementary material for: Population Pharmacokinetic Model of Iohexol in Dogs to Estimate Glomerular Filtration Rate and Optimize Sampling Time
Source: Front Pharmacol. 2021 Apr 29;12:634404. doi: 10.3389/fphar.2021.634404 (PMC8116701; doi:10.3389/fphar.2021.634404)
Supplement: Supplementary file 1 [file DataSheet1.docx]

Supplementary Material S1

**Population pharmacokinetic model of iohexol in dog to estimate glomerular filtration rate and optimize sampling time**

**Sarah Baklouti, Didier Concordet, Vitaliano Borromeo, Paola Pocar, Paola Scarpa^,^ Petra Cagnardi^*^.**

***Correspondence:** Petra Cagnardi: petra.cagnardi@unimi.it

# Materials and Method

# Supplementary Material on sampling protocol, iohexol analytical method and GFR estimation as reported in the previous paper by Pocar et al. 2019

**Iohexol injection and blood sampling**

The protocol was based on an already reported study by Lippi et al. (2008) with some modifications. Briefly, food was withheld from each dog for at least 12 hours before the procedure. Dogs were allowed free access to water throughout the study. Dogs were weighed and indwelling catheters were placed in the right and left cephalic veins. A commercially available iohexol formulation (Omnipaque®, Nycomed Amersham Sorin, Milan, Italy) was used. The nominal dose of iohexol was 64.7 mg/kg, and the exact dose was determined from the difference between the weights of the syringe before and after the injection. Iohexol was injected as a 60-s IV bolus into the catheter in the left cephalic vein. Two mL of blood were directly sampled from the right cephalic vein, transferred to a heparinized tube, and centrifuged at 2000×g for 15 min. Plasma was stored at −30°C until use. Samples were taken 5, 15, 60, 90, and 180 min after injection of the marker.

**Iohexol HPLC measurements**

Iohexol was determined using a Waters 626 HPLC system with a 996 photodiode array detector (Waters, Milford, MA, USA) (1 spectrum/s; wavelength 200-320 nm, extracting the chromatogram at 254 nm). Iohexol was separated in a Simmetry100 C18 column, 3.5 μm, 2.1 x 150 mm (Waters) using a mixture of CH_3_CN and 0.1% ortophosphoric acid in water (3:97, v/v) at a flow rate of 0.3 mL/min. During separation the column was held at 30°C. Standard iohexol (Omnipaque® 350, 755 mg/mL iohexol) was added to untreated dog plasma to obtain the following standard solutions: 5, 20, 50, 200 and 500 μg iohexol/mL. Plasma samples were deproteinized with 5% perchloric acid (1:1, v/v), centrifuged at 11000 g for 10 min at 5°C and 10 µL of supernatants were injected into the HPLC column. Data was processed using Millennium software (Waters). The peak areas of both iohexol isomers were used to calculate the iohexol concentrations and plasma clearance. The method was intra-laboratory validated. The specificity of the method was tested by analysing plasma samples before the iohexol injection. No interfering peaks were observed at the elution times as iohexol isomers. The limit of quantification was 1.80 μg/mL and the assay was linear over the concentration range of 5-500 μg/mL, with an average regression coefficient of 0.99 (n=22). For all calibration curves, the y-intercepts were virtually zero, indicating the absence of endogenous interferences. Precision, expressed as inter-day coefficient of variation (CV %), ranged from 4.4% to 7.8 % and the intra-day CV% from 3.2% to 5.9%. Accuracy ranged from 92% to 116%. For the quantification of iohexol in plasma the combined peak areas of the two isomers (exo- and endo-iohexol) were used.

**Calculation of glomerular filtration rate (GFR)**

GFR was determined by calculating the rate of iohexol clearance using Phoenix *WinNonlin*® software (version 8.0 - Certara L.P., St. Louis, MO, USA). Plasma clearance was determined with the following formula

$$Clearance=dose of iohexol injected/AUC$$

where AUC is the area under the curve calculated from plasma iohexol disappearance curves after an iv bolus.

GFR values were calculated by plotting the iohexol concentration against the sampling time for five samples (5, 15, 60, 90, and 180 min after iohexol) and AUC was calculated by the trapezoidal method with a non-compartmental pharmacokinetic model (linear log trapezoidal with extrapolation to inﬁnity). Clearance (mL/min) was normalized to body weight (BW) to obtain GFR, which was expressed as mL/min/kg.

**References**

Lippi I., V. Meucci, G. Guidi, G. Soldani (2008) Glomerular filtration rate evaluation in the dog throughout the plasmatic clearance of iohexol: simplified methods. Veterinaria; 22: 53-60.

**
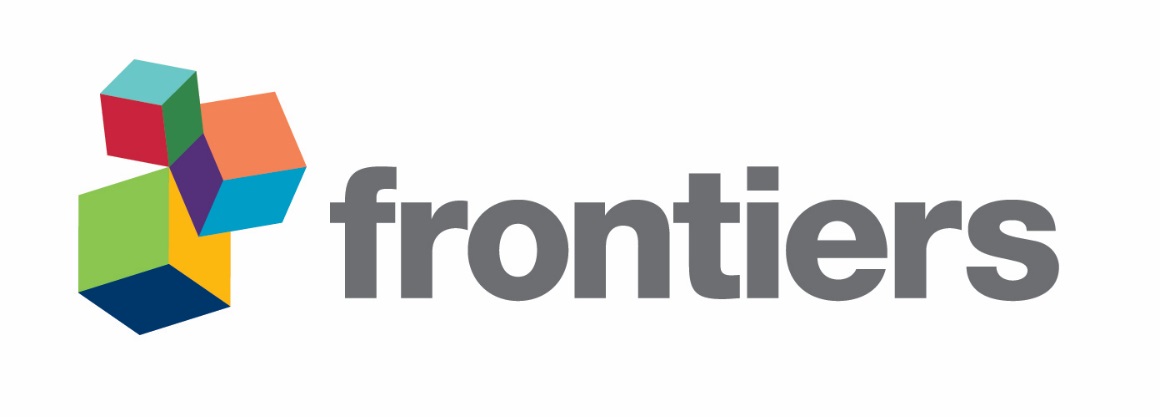
**
